# Uterine Injections in Endometritis

**Published:** 1870-08

**Authors:** 


Uterine Injections in Endometritis.—Dr. Nott, of New York (*Journal of Obst.*), writes a lengthy article on the treatment of endometritis by uterine injections. He strongly condemns the indiscriminate use of caustics and powerful stimulants to the inflamed and ulcerated uterus, as well as the almost sole reliance which not a few practitioners place on local treatment for uterine diseases.

His conclusions regarding the treatment of the disease in question are substantially as follows:

1. That the appalling symptoms sometimes following the use of uterine injections are induced by the passage of fluid through the Fallopian tubes into the peritoneal cavity, is decidedly questionable; they are more likely due to some effect produced upon the uterus.

2. If the fluid could always be prevented from over-filling the uterus, no such disaster would ever follow the use of an injection of proper strength; a free exit for the liquid would obviate the difficulty.

3. He has invented an instrument, which he figures, that will accomplish this purpose. It consists of a large catheter, within which is a small tube. The fluid is forced in through the little tube; it passes out an orifice into the uterus, and finds egress at once through the catheter, which it enters through ample slits.

Instead of sponge tents for dilating the os uteri, he uses a pair of curved dilating forceps, which act on the principle of the glove-stretcher. With this he makes forcible and rapid dilatation.

